# Effectiveness of the natural resistance management refuge for *Bt*-cotton is dominated by local abundance of soybean and maize

**DOI:** 10.1038/s41598-021-97123-8

**Published:** 2021-09-02

**Authors:** Benjamin Arends, Dominic D. Reisig, Shawnee Gundry, Anders S. Huseth, Francis P. F. Reay-Jones, Jeremy K. Greene, George G. Kennedy

**Affiliations:** 1 Department of Entomology and Plant Pathology, North Carolina State University, Campus Box 7630, Raleigh, NC 27695-7630 USA; 2 Department of Entomology and Plant Pathology, North Carolina State University, Vernon James Research and Extension Station, 207 Research Station Rd., Plymouth, NC 27692 USA; 3 Department of Plant and Environmental Sciences, Clemson University, Pee Dee Research and Education Center, 2200 Pocket Road, Florence, SC 29506 USA; 4 Department of Plant and Environmental Sciences, Clemson University, Edisto Research and Education Center, 64 Research Road, Blackville, SC 29817 USA

**Keywords:** Biotechnology, Ecology, Evolution, Plant sciences, Ecology

## Abstract

Genetically engineered crops expressing *Bacillus thuringiensis* (*Bt*) Cry toxins have transformed insect management in maize and cotton, reducing insecticide use and associated off-target effects. To mitigate the risk that pests evolve resistance to *Bt* crops, the US Environmental Protection Agency requires resistance management measures. The approved resistance management plan for *Bt* maize in cotton production regions requires a structured refuge of non-*Bt* maize equal to 20% of the maize planted; that for *Bt* cotton relies on the presence of an unstructured natural refuge comprising both non-*Bt* crop and non-crop hosts. We examined how abundance of *Bt* crops (cotton and maize) and an important non-*Bt* crop (soybean) component of the natural refuge affect resistance to *Bt* Cry1Ac toxin in local populations of *Helicoverpa zea*, an important lepidopteran pest impacted by *Bt* cotton and maize. We show refuge effectiveness is responsive to local abundances of maize and cotton and non-*Bt* soybean, and maize, in its role as a source of *H. zea* infesting cotton and non-*Bt* hosts, influences refuge effectiveness. These findings have important implications for commercial and regulatory decisions regarding deployment of *Bt* toxins targeting *H. zea* in maize, cotton, and other crops and for assumptions regarding efficacy of natural refuges.

## Introduction

The widespread adoption of genetically engineered crops expressing insecticidal toxins from *Bacillus thuringiensis* (*Bt*) has transformed insect management, especially in cotton (*Gossypium hirsutum* L.) and maize (*Zea mays* L.), resulting in reduced insecticide use and associated off-target effects, enhanced yields, and increased farmer profits^1–6^. In 2020, *Bt* varieties accounted for 88 and 82% of the cotton and maize, respectively, planted in the US^7^. This widespread adoption has resulted in area-wide suppression of some targeted pest populations and benefits that transcend *Bt* crops^8–10^. Resistance management measures are mandated by the US Environmental Protection Agency [EPA] as a condition for registration of *Bt* crops to preserve their associated crop protection and broader environmental benefits. Nonetheless, the benefits of *Bt* technology in maize and cotton are threatened by the evolution of resistance in several key pest species^11–16^. Principal among these in the southern US is *Helicoverpa zea* (Boddie) (Lepidoptera: Noctuidae)*,* a consistent pest of both cotton and maize, commonly referred to as bollworm or corn earworm. Field-evolved *H. zea* resistance to *Bt* crystal-forming (Cry protein) toxins in maize and cotton has resulted in increasing damage in both crops and an increase in insecticide use in cotton and sweet corn^11,13,14^.

Resistance management for *Bt* crops relies on the presence of a refuge from selection for resistance comprising non-*Bt* host plants. To function effectively, the refuge should be spatially close to the *Bt* crop and concurrently produce enough *Bt-*susceptible insects to mate with *Bt*-resistant individuals emerging from the *Bt* crop, thereby minimizing the frequency of resistance alleles in the population^12^. This strategy is most effective when *Bt*-plants express the toxin at a concentration sufficient to kill individuals heterozygous for a recessive resistance allele; generally referred to as high dose^12,17^. Although *Bt* maize hybrids expressing a single *Bt* toxin continue to be planted in limited areas, *Bt* maize and cotton varieties expressing multiple *Bt* toxins (pyramids) have replaced single-toxin varieties. Insects resistant to one of the toxins in a pyramid can be killed by the other(s). Pyramids are most effective when each toxin is expressed at a high dose and there is no cross-resistance between toxins^12^. Concentrations of Cry toxins expressed in *Bt* maize and cotton are high dose for some targeted lepidopteran pests (e.g., stalk borers in maize; tobacco budworm and pink bollworm in cotton) but not for *H. zea*, increasing the speed with which resistance is expected to develop^12^.

The US EPA-approved resistance management plan for *Bt* maize in cotton production regions requires planting a structured refuge of non-*Bt* maize equal to 20% of the total maize planted. In contrast, the refuge portion of the resistance management plan for *Bt* cotton relies on an unstructured natural refuge comprising non-*Bt* crop and non-crop host plants present in the refuge landscape^12,18^. Because the host range of *H. zea* encompasses many of the crops and non-crop plant species that are abundant in cotton production systems of the southeastern USA, it is assumed that the diversity and abundance non-*Bt* host plants in the natural refuge will produce enough *Bt* susceptible *H. zea* moths at the appropriate time to function effectively as a refuge. This assumption was critically examined by a Scientific Advisory Panel in 2006^19^ and supported by studies that have shown *H. zea* populations developing on cotton were a relatively small proportion of the total population^20,21^. In 2018, a Scientific Advisory Panel^18^ recommended continued use of the natural refuge strategy for managing *H. zea* resistance to *Bt* toxins expressed in cotton.

Because the speed of resistance evolution is inversely related to the amount of refuge^22^, understanding factors influencing how the natural refuge functions for *H. zea* in cotton production systems is critical to inform development of resistance management strategies and regulatory policies relating to their implementation. Herein, we examine effects of varying abundance of two *Bt* crops (cotton and maize) and non-*Bt* soybean (*Glycine max* L.) within local landscapes in commercial field crop production systems on effectiveness of the natural refuge in suppressing resistance in *H. zea* populations to a *Bt* toxin (Cry1Ac). Soybean is an important non-*Bt* crop host of *H. zea* that varies greatly in abundance among locations.

### *Helicoverpa zea *and the *Bt* maize and cotton production system

*Helicoverpa zea* is a highly polyphagous, multivoltine pest that has numerous crop and non-crop hosts^23,24^. In the southeastern US, *H. zea* can complete at least four generations per year, with each generation potentially feeding on different crops at various phenological stages^25^. Adult moths that developed on maize ears disperse to infest other suitable crop and non-crop host plants. Included among these are bloom-stage cotton, soybean, peanut, and sorghum^25,26^. The importance of these crops as hosts following dispersal from maize varies greatly across the cotton production region, with soybean being particularly important in North Carolina^26^. Selection for resistance to *Bt* toxins occurs almost exclusively on *Bt* maize and cotton. However, maize also serves as an important source of *H. zea* that subsequently infest cotton, soybean and other non-*Bt* host plants comprising the natural refuge^25,26^. In cotton production areas, the non-*Bt* structured refuge required for *Bt* maize^12,21^ is a potentially important source of susceptible *H. zea* that subsequently infest cotton and the natural refuge for *Bt* cotton. However, compliance with the structured refuge requirement for maize by growers has been problematic^12,27^.

Cry1Ac, in combination with one or more other *Bt* toxins, is currently expressed in all *Bt* cotton varieties; hence, populations developing on cotton are selected for resistance to Cry1Ac. Cry1Ac is not expressed in maize but the closely related Cry1Ab and Cry1A.105 toxins, also active against *H. zea*, are found in combination with other Cry toxins in commonly grown maize varieties. Cross resistance between Cry1Ac and Cry1Ab has been documented, so it is expected that indirect selection for resistance to Cry1Ac occurs in maize that expresses Cry1Ab^11,12,21,28,29^. Genetically modified soybean expressing *Bt* toxins is not registered for use in the US.

A majority of *H. zea* larval populations that complete development in cotton and soybean subsequently overwinter as pupae in the soil. Previous research documented the importance of soybean as a late-season host for *H. zea* in North Carolina^26^. Based on this biology, we tested the assumption that underlies the natural refuge strategy for cotton; namely that the abundance and diversity of non-*Bt* crop and non-crop host plants in the local landscape are sufficient in practice to ensure the presence of a functional natural refuge. Specifically, we hypothesized that effectiveness of the natural refuge in suppressing Cry1Ac resistance in *H. zea* is dependent on the relative abundances of cotton and soybean in the local landscape. Because maize acts as a selection site for Cry-toxin resistance and a source of selected and non-selected populations (the latter originating from the structured, non-*Bt* maize refuge) infesting both cotton and the natural refuge, we hypothesized that effects of the relative abundances of cotton and soybean on resistance of local *H. zea* populations are also dependent on the relative abundance of maize in the local landscape.

To investigate these hypotheses, we measured survival of larval offspring of *H. zea,* collected from non-*Bt* maize at 59 field locations in North and South Carolina, following exposure to a diagnostic concentration of the Cry1Ac toxin. Because resistance levels of *H. zea* to Cry1Ac have been shown previously to vary greatly among local populations of *H. zea*^14^, we expected that selection occurring locally and in the most recent past would have a strong influence on larval survival in the bioassay. We examined the relationships between the abundances of maize, cotton, and soybean within a 1-km radius of each collection site during the preceding year and effectiveness of the natural refuge as measured by variation in larval survival in the bioassay. Larval survival was fit to a binomial distribution with random effects intercepts for sample year using a generalized linear mixed model. Independent variables included proportional areas of each crop and their respective two-way interactions (cotton * maize, maize * soybean, and cotton * soybean).

## Results and discussion

### Resistance of H. zea populations to Cry1Ac

**Figure 1:**
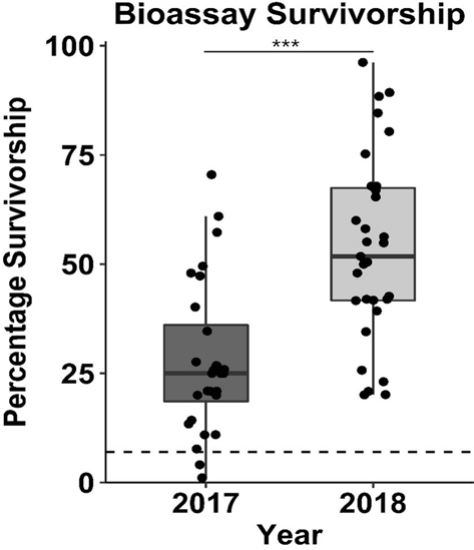
Survival of *H. zea* larvae collected in 2017 and 2018 following exposure to 29 µg/cm^2^ Cry1Ac in diet-overlay assay. Dashed line is mean survival of a known susceptible field population. ***Years significantly different *F* = 25.38; df = 1, 58; *P* < 0.0001.

To measure variation in resistance of *H.* zea populations across field locations during 2017 and 2018, larval offspring of insects collected from non-*Bt* maize were subjected to a diet-overlay bioassay containing a diagnostic concentration of Cry1Ac (29 µg/cm^2^) corresponding to the mean LC_95_ of four Cry1Ac susceptible *H. zea* populations. Overall, larval survivorship varied significantly among years (Fig. 1).

**Figure 2:**
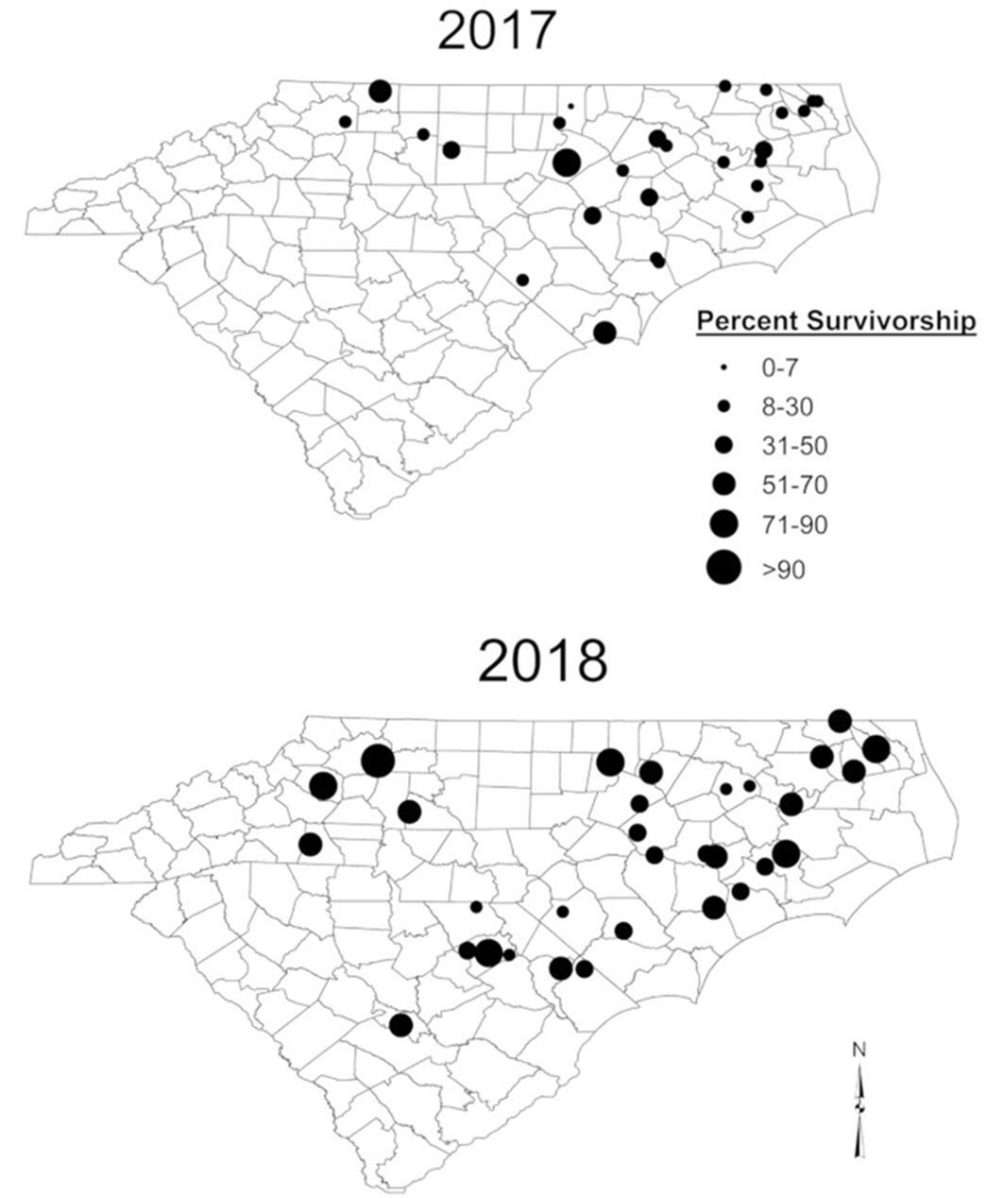
Survivorship of *H. zea* from different collection sites in 2017 (n = 28) and 2018 (n = 31) following exposure to 29 µg/cm^2^ Cry1Ac in diet-overlay assay. Each dot represents the collection site and dot size represents percent survivorship.

The wide range in survivorship (1 to 96%) among locations reveals high levels of spatial variation in resistance of local *H. zea* populations to the Cry1Ac toxin (Fig. 2). Because the populations included in our study were collected as larvae from ears of non-*Bt* maize and their offspring were subjected to the bioassay, the response did not reflect effects of selection for resistance on the parents of larvae used in our bioassays.

### Effects of prior year cotton, maize, and soybean abundance on Cry1Ac resistance

In our bioassay, higher survival indicates higher resistance to Cry1Ac. Selection for resistance to *Bt* toxins in *H. zea* occurs almost exclusively in maize and cotton, and selection is reduced at higher levels of relative abundance of non-*Bt* hosts in the landscape. The relationships are complex, reflecting how differences in the relative abundance of each of these crops affect *H. zea* populations and their associated inter-crop source-sink dynamics. These dynamics, in turn, influence the intensity of selection on the local populations.

The main effects of proportional areas of cotton and maize on *H. zea* survival in the bioassay are highly significant (P < 0.0003 and P < 0.0001, respectively) but neither the main effect of soybean nor the cotton * soybean interaction effect is significant (P = 0.2429 and P = 0.0967, respectively). Importantly, the cotton * maize and the maize * soybean interaction effects are both highly significant (P < 0.0001; Supplementary Table S1).

**Figure 3:**
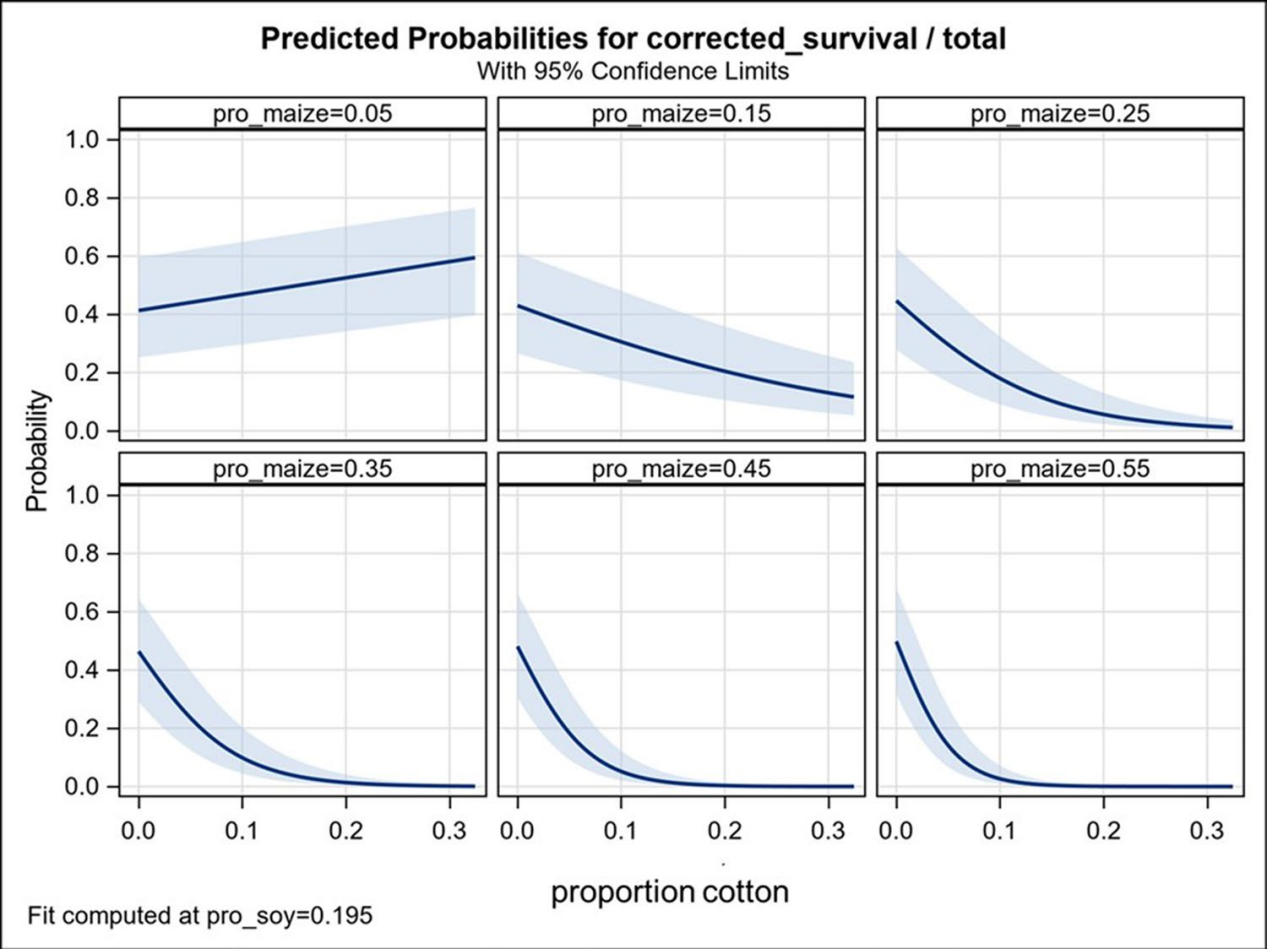
Predicted survival of *H. zea* larvae in response to proportional areas of cotton at six levels of proportional abundance of maize during the prior year within a 1-km radius of the collection site. Proportional abundances of maize shown (0.05; 0.15; 0.25; 0.35; 0.45; 0.55). were selected to illustrate changes in response to maize abundance across the range observed in our study (0.01 to 0.54). Data provided in Table S2. Fit is computed at the mean proportional area of soybean (0.195). Shaded bands are 95%CI.

To examine the cotton * maize interaction, probability of survival was fit to the abundance of cotton at different levels of maize abundance and at the mean abundance of soybean within a 1-km radius of the collection site during the prior year (Fig. 3). The result shows a small increase in larval survival in response to increases in abundance of cotton when maize abundance is very low (0.05), but a negative relationship between larval survival and cotton abundance that becomes increasingly strong as maize abundance increases. The latter can be interpreted to indicate that effectiveness of the local natural refuge increases with increasing abundance of maize. Ineffectiveness of the local refuge when proportion of maize was low (0.05) likely results from low maize abundance limiting the local *H. zea* population early in the season, resulting in an increase in the relative proportion of the population that immigrated to the study area after being subjected to selection elsewhere.

**Figure 4:**
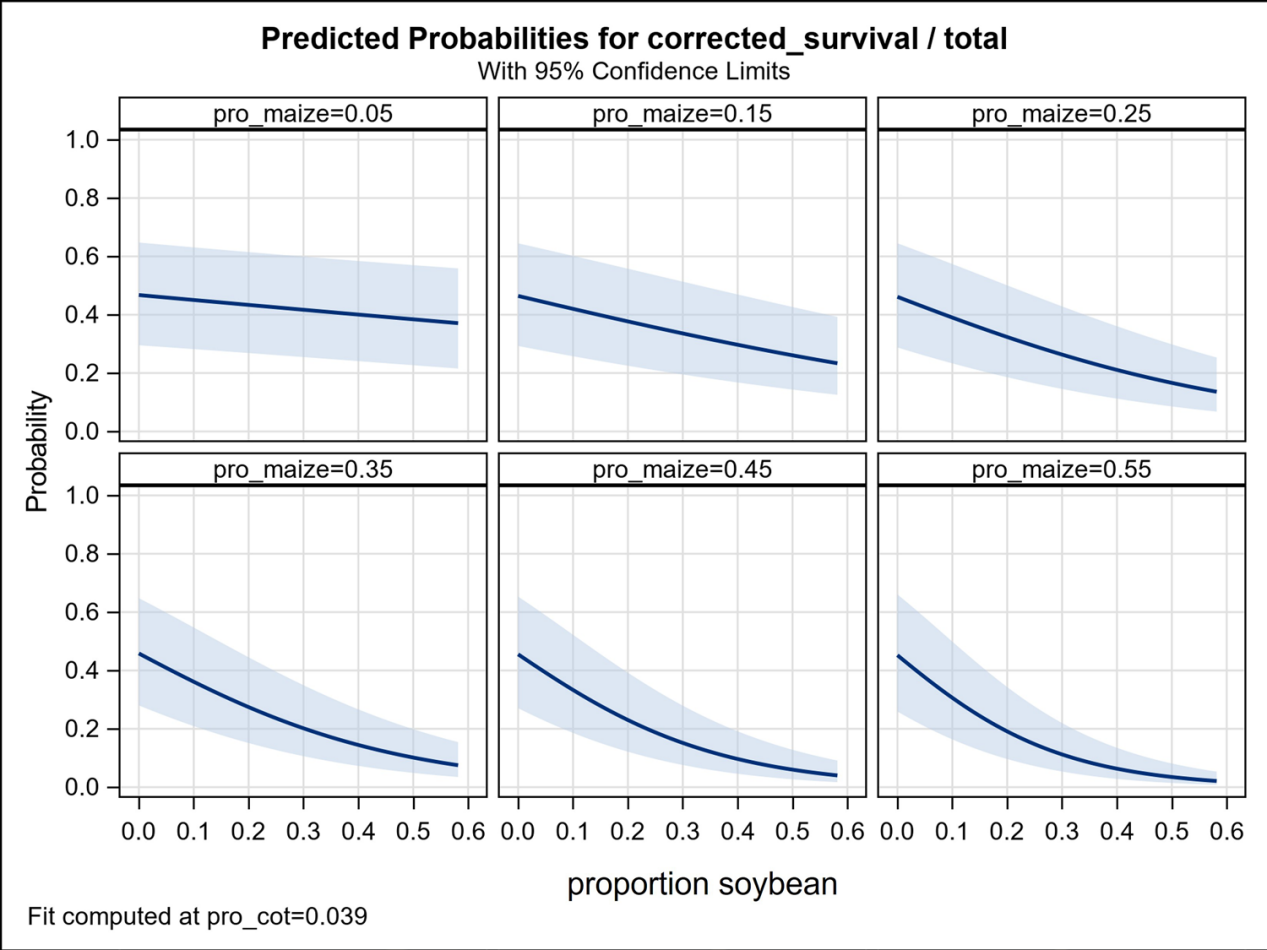
Predicted survival of *H. zea* larvae in response to proportional areas of soybean at six levels of proportional abundance of maize during the prior year within a 1-km radius of the collection site. Proportional abundances of maize shown (0.05; 0.15; 0.25 0.35; 0.45; 0.55) were selected to illustrate changes in response to maize abundance across the range observed in our study (0.01 to 0.54). Data provided in Table S2. Fit is computed at the mean proportional area of cotton (0.039). Shaded bands are 95%CI.

The role of soybean as the primary constituent of the natural refuge is revealed by the maize * soybean interaction in which larval survival is largely independent of soybean abundance in the local landscape at low levels of maize abundance but shows an increasingly strong negative response as maize abundance increases (Fig. 4). Together these results indicate that the effect of proportional area of any one of either maize, cotton, or soybean on the resistance level of the local *H. zea* population is dependent on the proportional areas of the other two.

Maize abundance strongly influences the size of the dispersing *H. zea* populations that infest subsequent hosts based on their relative attractiveness and availability in the local landscape^25,26,30^. Cotton and soybean are most attractive to *H. zea* when flowering, which typically coincides with moth dispersal from maize. Flowering soybean is more attractive than flowering cotton^31^. Although populations that develop on soybean are influenced by numerous factors^32^, larval populations infesting soybean in North Carolina are much higher than those infesting cotton^26^. Hence, the number of susceptible moths produced per unit area on soybean is expected to exceed that of resistant moths completing development on cotton^20^. Populations infesting cotton are not only selected for resistance to *Bt* toxins but are also increasingly targeted by insecticide applications to reduce damage by *Bt*-resistant larvae^14^. To the extent that these applications reduce the size of *H. zea* populations under selection for Cry toxin resistance in cotton, they can be expected to increase the effective size of the natural refuge. The contribution of soybean to the natural refuge for *Bt* Cry toxins in cotton varies among locations depending on the relative abundances of maize, cotton, and soybean in the local landscape. We did not specifically consider other crop and non-crop hosts of *H. zea* in the landscape, but other hosts contributing to the natural refuge are represented in the proportional abundance within the 1-km buffer not occupied by maize, cotton, and soybean. Our results indicate that although soybean is only one component of the natural refuge, variation in soybean abundance is an important determinant of effectiveness of the natural refuge across the sites studied.

The effect of maize abundance on natural refuge efficacy may be explained by its dual role as a site for selection for cross resistance among Cry1 toxins^11,12,21,28,29^ and as a nursery producing moths that disperse from maize fields to infest cotton, soybean, and other plants comprising the natural refuge^25,26,30^. *Helicoverpa zea* moths produced on maize include those subjected to selection on *Bt* varieties, as well as susceptible moths that developed on non-*Bt* varieties planted to meet structured-refuge requirements for *Bt* maize. Increasing effectiveness of the natural refuge for *Bt* cotton with increasing maize abundance points to the importance of the structured refuge for *Bt* maize as a key source of Cry-toxin susceptible moths dispersing into the natural refuge for *Bt* cotton. Our results suggest that poor compliance by growers with the structured refuge requirement for maize, which is widespread^12,27^, not only compromises its effectiveness, but also undermines effectiveness of the natural refuge for *Bt-*cotton.

Before maize and cotton expressing pyramided toxins were adopted beginning in 2007, an unstructured natural refuge was considered inadequate for single-toxin cotton, and a structured refuge was required. The current US EPA approved resistance management plan for *Bt* cotton relies on expression of multiple toxins, which are not high dose against *H. zea,* and the presence of an unstructured natural refuge^12,21^. Implicit in the rationale for a natural refuge is that the total abundance and diversity of non-*Bt* crops and natural hosts remain sufficiently stable over time and space to ensure the refuge is functional. A Scientific Advisory Panel, convened in 2006 by the US EPA^19^, critically examined the feasibility of a natural refuge for *Bt-*cotton and recognized the potential influence of variation in abundance of non-*Bt* crop hosts within the natural refuge on the effective size of the refuge for *H. zea,* but empirical studies documenting this relationship have been lacking*.* The Panel also recognized that this variation might differ in importance among cotton production regions. Current levels of sensitivity of *H. zea* populations to Cry1Ac have likely been influenced by selection over many years, including that by single-gene Cry1Ac cotton and Cry1Ab maize, and more recently by selection and cross-resistance among related pyramided toxins in maize and cotton. Our findings indicate that on-going selection is important and that despite the capacity of *H. zea* for long-distance dispersal^24^, the effects of local abundance of soybean in relation to maize and cotton, acting within the larger natural refuge, influence the resistance levels of local *H. zea* populations at least two generations later in the following growing season.

Because abundance of soybean as a component of the natural refuge varies among US cotton production regions^26^, the relationships we observed for soybean are expected to vary in importance among regions as well. However, we believe the implications of our findings are general. They indicate that effectiveness of natural refuges can be expected to vary among locations and years in response to differences in local abundances of relevant *Bt* and non-*Bt* crop hosts of *H. zea*, and that maize as a source of *H. zea* infesting cotton and non-*Bt* hosts in the landscape can be especially influential in determining natural refuge effectiveness. Examination of the proportional abundances of maize, cotton, and soybean in a 1-km radius surrounding our sample sites over a 5-year period encompassing our study (2014–2018) reveals considerable variation in abundances among years as well as among locations (Supplementary Table S3). Given the availability of geospatial crop production data, trends in relative crop composition could be leveraged to better understand where crop components of unstructured natural refugia could be manipulated to improve *Bt* toxin resistance management in economically important polyphagous pests, like *H. zea.*

These findings have important implications for commercial and regulatory decisions regarding potential future deployment of *Bt* Cry toxins in soybean and *Bt* Vip3 toxins targeting *H. zea* in maize, cotton, and potentially other crops^12,21^, as well as for assumptions regarding the efficacy of natural refuges for resistance management. They provide evidence that in the North and South Carolina field crop production systems studied, effectiveness of the natural refuge for cotton depends on the abundance of soybean as the dominant agricultural component of the natural refuge and on the relative abundance of maize in the local landscape. This dependency suggests that refuge requirements for *Bt* maize targeting *H. zea* in cotton production areas should reflect the important role of maize as a source for the *H. zea* populations that are critical to the functioning of the natural refuge.

## Methods

### Characterizing resistance of H. zea populations to Cry1Ac

In 2017 and 2018, *H. zea* larvae were collected from plots of non-*Bt* maize hybrid Dekalb 67-70RR grown in maize-growing counties across North Carolina and South Carolina from seed provided by Bayer Crop Science, St. Louis, MO, US. Plots four to twelve rows wide and > 40 m long were maintained following agronomic recommendations from the local Cooperative Extension Service. Spatial coordinates were recorded at each location using a handheld GPS device. There were 28 plots in North Carolina in 2017 and 24 and 7 plots in North and South Carolina in 2018, respectively. Most plots were grown on commercial farms. All aspects of this study were conducted in compliance with institutional, local, and national regulations and permission to grow and collect leaf and insect samples was obtained from the landowners. Leaf samples from each of three randomly selected plants per plot were tested for presence of Cry1A toxin using an ELISA strip test (QuickStix Kit for Cry1Ab Maize Leaf & Seed, Envirologix Inc., Portland, ME) to verify the plots were non-*Bt* maize.

Fifty to 120 larvae were collected from ears randomly selected from plants at least 5 m from the ends of the middle rows of each plot. Larvae were immediately placed on artificial diet in 30 mL plastic cups sealed with cardboard lids and held in coolers during transport to the laboratory where they were reared to pupation. The commercial *H. zea* diet (Southland Products, Lake Village, AR, USA) was modified by adding casein to achieve a protein:carbohydrate ratio of 1.6:1 and supplemented with agar, anti-microbials and cellulose^33^. Pupae of each population were surface sterilized in a 1.3% bleach solution and placed in 1.8 L containers at a 3:1 female to male ratio (maximum 28 pupae per container). Containers were covered with cheesecloth to provide a substrate for oviposition and maintained at 25 °C, 50% RH, and natural photoperiod. Upon eclosion, moths were provided with 10% sucrose solution *ad libitum*. Eggs collected from the cheesecloth were transferred to 0.5-L containers where they hatched. Neonates less than 24 h old from each collection were used in a diet-based diagnostic-dose bioassay.

Bioassays were prepared by adding 0.75 mL of diet to each well of a 128-well plastic tray, which was then covered and refrigerated until used. Diet trays were warmed to room temperature prior to overlaying the diet in each of 112 wells per tray. A 40 µL aliquot of a solution of Cry1Ac protein (94–96% pure, trypsin activated, ion exchange HPLC purified, desalted, and freeze dried, purchased from Case Western Reserve University) dissolved in Triton X-100 (0.1%) buffer was applied to the diet surface to produce a Cry1Ac dose of 29 µg/cm^2^, corresponding to the mean LC_95_ of four Cry1Ac susceptible *H. zea* colonies. Three colonies were started from field collections from Mississippi, North Carolina, and Louisiana in 2017, 2016, and 2016, respectively, and subsequently maintained in the laboratory at NC State University. The fourth was a laboratory colony obtained from Benzon Research, Inc. (Carlisle, PA, USA). In multiple bioassays of the Louisiana colony, this concentration resulted in a mean survivorship of 7% (±4.8 SD; n=4)^34^. Diet overlaid with 100 µL of aqueous Triton X-100 buffer in each of the remaining 16 cells per tray served as a control. Once the Cry1Ac solution dried, one neonate per well was added using a fine-tipped brush. The trays were then covered with a self-adhesive plate. Because colony size varied, not all assays had 112 larvae in the treated wells, but all had 16 larvae in the control wells. In 2017, bioassays were incubated in a growth chamber (27 °C, 60% RH, 14:10 photoperiod). In 2018, they were incubated at 25 °C and 50% RH to avoid condensation associated with a manufacturing change in cover plates. Bioassays were held for 7 days, after which mortality was assessed. Larvae that did not move after prodding with a brush were scored as dead. Proportion survival was corrected for control mortality using Abbott’s method^35^. Although our mortality measure did not include “functionally dead” larvae, we believe it provides a meaningful measure of variation in sensitivity to Cry1Ac among locations included in the study. We base this on the consistency of mortality among our reference populations and the large variation in survivorship we observed among the field collected populations in our study (ranging from 1% to 100% survival at 7 days). This belief is further strengthened by our finding that variation in bioassay response is related to variation in relative abundances of maize, cotton, and soybean in a biologically meaningful way.

### Characterizing effects of prior year cotton, maize, and soybean abundance on Cry1Ac resistance

Larvae were collected from non-*Bt* maize plots to avoid confounding effects of resistance selection during the current year on survival. This allowed us to test for effects of maize, cotton and soybean abundance on resistance selection occurring during the prior year. Because no estimates of actual acreages of *Bt* maize and *Bt* cotton in landscapes surrounding each of the sample locations were available, our analysis was based on acreages of total maize, cotton, and soybean. Nationally, *Bt* varieties accounted for 88 and 82% of the cotton and maize, respectively, planted in the US during 2020^7^. Landscape composition surrounding each sample site was determined using remotely sensed data from the USDA National Agricultural Statistics Service-Cropland Data Layer (CDL)^36^. Based on findings that a majority of marked *H. zea* moths emerging from maize fields were captured within 0.8 km of the source field^37^, we assumed that selection for resistance occurred locally on maize and cotton, and the abundance of these two crops would have the strongest effect on resistance levels observed in our samples. Therefore, we calculated proportional areas of maize, cotton, and soybean during the prior growing season within a 1-km buffer surrounding each collection location using ArcGIS (ESRI 2018, Redlands, CA).

Prior to analyzing relationships between larval survival and abundance of maize, cotton, and soybean, we tested for relationships between the proportional areas of these crops within the buffer using linear regression in SAS version 9.4^38^. Proportional areas of cotton and soybean, and of cotton and maize, were not related. The proportional areas of maize and soybean were weakly but positively related; the regression accounted for only 7.2% of total variation (Supplemental Table S4).

To examine the relationships between the abundances of maize, cotton and soybean in the local landscape and effectiveness of the natural refuge as measured by variation in larval survival in the bioassay, we used a generalized linear mixed model with a binomial distribution and a logit link function in the GLIMMIX procedure of SAS version 9.4^38^. Based on a separate analysis of variance demonstrating significant differences in bioassay response between years, the analysis included sample year as random effect intercepts. The model reflected the seasonal dynamics of *H. zea* populations and the ways in which maize, cotton and soybean are expected to influence selection for resistance. Independent variables included proportional areas of each of the crops and their respective two-way interactions (cotton * maize, maize * soybean and cotton * soybean). This model was selected over others that included the 3-way interaction because it had the lowest AIC value and allowed us to generate confidence intervals for predicted probability of survival. A Moran’s I test was conducted in R version 3.6.0^39^ to test for spatial autocorrelation among sample sites^40^. Using survival in bioassays as the predictor, there was no evidence of autocorrelation among sample sites (P = 0.29), indicating that bioassay survival among sample sites was independent.

## Supplementary Information

Supplementary Information.
