# Supplementary material for: Effectiveness of the natural resistance management refuge for Bt-cotton is dominated by local abundance of soybean and maize
Source: Sci Rep. 2021 Sep 2;11:17601. doi: 10.1038/s41598-021-97123-8 (PMC8413434; doi:10.1038/s41598-021-97123-8)
Supplement: Supplementary file 1 — Supplementary Information. [file 41598_2021_97123_MOESM1_ESM.pdf]

Supplementary Information for

Effectiveness of the natural resistance management refuge for *Bt*-cotton is dominated by local abundance of soybean and maize

Benjamin Arends, Dominic D. Reisig, Shawnee Gundry, Anders S. Huseth, Francis P. F. Reay-Jones, Jeremy K. Greene, George G. Kennedy

Paste corresponding author name: **George G. Kennedy[1]**
Email: gkennedy@ncsu.edu

This PDF file includes:

Table S1
Table S2
Table S3
Table S4

Supplementary Table S1. Generalized Linear Mixed Model estimates of effects of proportional areas of maize, cotton, soybean, and their respective interactions within a 1-km radius of collection site on probability of survival of *H. zea* larvae following exposure to 29 µg/cm^2 Cry1Ac in diet-overlay bioassay.[1]

Effect	Estimate	SE	DF	T value	P>[t]
Intercept	-0.3012	0.3798	1	-0.79	0.5732
Maize	2.8560	0.4304	51	6.64	<.0001
Cotton	4.5465	1.1624	51	3.91	0.0003
Soybean	-0.4309	0.3647	51	-1.18	0.2429
Maize* Cotton	-76.3099	8.6708	51	-8.80	<0.0001
Maize*Soybean	-11.1448	1.7265	51	-6.46	<0.0001
Cotton*Soybean	7.8423	4.6346	51	1.69	0.0967

[1]Year included as a random effect in the analysis.

Supplementary Table S2. **Site, year, areas and proportional areas of cotton, maize, and soybean within a 1-km radius of each *Helicoverpa zea* larval collection site; numbers of larvae and numbers of surviving larvae in the control and corresponding Cry1Ac treatments for each bioassay, and Abbott's corrected larval survival in each bioassay.[35]**

Year	hectares cotton	proportional area cotton	hectares corn	proportional area corn	hectares soybean	proportional area soybean	control number larvae	control number survivors	bioassay number larvae	bioassay number survivors	bioassay Abbotts corrected survival
2017	0	0	19.9001	0.0633	16.3883	0.0522	16	0	112	28	28
2017	0	0	85.9038	0.2734	13.4168	0.0427	16	1	112	64	68
2017	0	0	94.7282	0.3015	3.4217	0.0109	16	1	112	21	22
2018	0	0	93.3776	0.2972	0.4502	0.0014	16	1	112	101	108
2017	0	0	94.9984	0.3024	169.5564	0.5397	16	3	112	7	9
2017	0	0	20.9807	0.0668	28.9047	0.0920	16	1	112	52	55
2017	0	0	8.1041	0.0258	11.7960	0.0375	16	3	112	1	1
2017	0	0	11.1657	0.0355	6.4833	0.0206	16	0	64	7	7
2018	0	0	12.2462	0.0390	8.5544	0.0272	16	0	112	100	100
2018	0	0	10.4453	0.0332	18.2793	0.0582	16	2	112	54	62
2018	0	0	13.6870	0.0436	15.7580	0.0502	16	0	112	58	58
2017	0	0	15.8481	0.0504	41.8713	0.1333	16	0	96	55	55
2018	0	0	36.5586	0.1164	17.5589	0.0559	16	0	112	75	75
2018	0	0	4.3222	0.0138	0.0000	0.0000	16	0	112	99	99
2017	0	0	11.0756	0.0353	22.8716	0.0728	16	0	64	7	7
2017	0.0900	0.0003	120.1212	0.3824	182.7931	0.5818	16	0	112	15	15
2017	0.0900	0.0003	23.2318	0.0739	9.4548	0.0301	16	1	112	74	79
2017	0.0900	0.0003	165.0541	0.5254	68.7050	0.2187	16	1	112	27	29
2017	0.1801	0.0006	62.5819	0.1992	128.9457	0.4104	16	0	112	16	16
2018	0.1801	0.0006	4.0521	0.0129	28.3644	0.0903	16	4	112	35	47
2017	0.3602	0.0011	0.0900	0.0003	37.5491	0.1195	16	1	112	22	23
2018	0.3602	0.0011	25.8432	0.0823	43.2220	0.1376	16	1	112	61	65
2017	0.3602	0.0011	27.4640	0.0874	46.0134	0.1465	16	0	112	29	29
2017	0.4502	0.0014	60.5108	0.1926	87.8848	0.2797	16	0	112	45	45
2017	0.7204	0.0023	64.2027	0.2044	48.2646	0.1536	16	0	112	30	30
2018	0.7204	0.0023	105.4437	0.3356	43.4922	0.1384	16	1	75	35	38
2018	0.9905	0.0032	169.1962	0.5386	92.7472	0.2952	16	0	112	44	44

2017	1.2606	0.0040	116.8795	0.3720	84.1028	0.2677	16	1	112	22	23
2018	1.4407	0.0046	8.7344	0.0278	39.8903	0.1270	16	2	71	21	25
2018	1.6208	0.0052	1.8009	0.0057	145.9643	0.4646	16	1	53	21	23
2018	2.4312	0.0077	57.4493	0.1829	64.8330	0.2064	16	2	112	47	54
2018	3.1516	0.0100	38.3595	0.1221	128.8556	0.4102	16	3	112	77	95
2018	3.6018	0.0115	33.7672	0.1075	95.8988	0.3053	16	0	82	45	45
2018	3.6018	0.0115	27.5540	0.0877	61.8615	0.1969	16	1	112	63	67
2018	4.6824	0.0149	6.2132	0.0198	144.1634	0.4589	16	1	80	31	33
2018	4.7724	0.0152	42.7718	0.1361	111.6569	0.3554	16	3	98	16	20
2018	5.1326	0.0163	55.6483	0.1771	30.5255	0.0972	16	0	112	47	47
2017	5.4028	0.0172	4.0521	0.0129	94.9984	0.3024	16	0	112	53	53
2018	5.6729	0.0181	3.7819	0.0120	21.1608	0.0674	16	3	112	19	23
2017	5.7629	0.0183	0.9905	0.0032	85.2734	0.2714	16	3	112	19	23
2017	5.9430	0.0189	86.0838	0.2740	76.9892	0.2451	16	2	112	47	54
2017	12.3363	0.0393	68.7050	0.2187	168.3858	0.5360	16	2	112	4	5
2017	14.3173	0.0456	30.9758	0.0986	47.3641	0.1508	16	1	80	20	21
2018	17.7390	0.0565	61.5914	0.1961	36.2885	0.1155	16	0	112	76	76
2018	19.4499	0.0619	32.1464	0.1023	91.5766	0.2915	16	1	112	79	84
2017	23.0517	0.0734	49.3451	0.1571	70.5059	0.2244	16	1	112	21	22
2017	27.0138	0.0860	17.6490	0.0562	74.6480	0.2376	16	8	75	13	26
2017	29.0848	0.0926	39.3500	0.1253	35.7482	0.1138	16	1	112	29	31
2018	32.7767	0.1043	17.0187	0.0542	67.9846	0.2164	16	0	112	63	63
2018	34.0373	0.1083	60.2407	0.1918	136.6896	0.4351	16	0	78	18	18
2018	36.4686	0.1161	40.3405	0.1284	45.8333	0.1459	16	1	112	27	29
2017	37.0088	0.1178	46.5537	0.1482	65.3733	0.2081	16	0	112	28	28
2018	37.7292	0.1201	2.3412	0.0075	1.1706	0.0037	16	0	112	76	76
2018	37.8193	0.1204	5.6729	0.0181	70.0557	0.2230	16	1	112	53	57
2018	41.0609	0.1307	32.1464	0.1023	14.6775	0.0467	16	0	112	47	47
2017	44.5727	0.1419	19.8101	0.0631	134.8887	0.4294	16	0	112	28	28
2018	45.0229	0.1433	51.9565	0.1654	49.2551	0.1568	16	1	106	20	21
2018	72.6670	0.2313	18.2793	0.0582	50.4257	0.1605	16	8	112	45	90
2018	102.022	0.3247	27.3739	0.0871	65.4633	0.2084	16	1	62	38	41

[35] W.S. Abbott, A Method of Computing the Effectiveness of an Insecticide. *J. Am. Mosq.* 3(2), 302–303 (1925).

Supplementary Table S3. Mean and standard deviation of proportional abundance of cotton, maize, and soybean within a 1-Km radius of each *H. zea* collect site from 2014 through 2018. *Year indicates year larvae were collected for bioassay. **Larvae were collected for bioassay in both 2017 & 2018.

Year*	State	Site	Cotton	Maize	Soybean
2017	North Carolina	2	0.034 (0.033)	0.185 (0.057)	0.236 (0.04)
		3	0.171 (0.049)	0.079 (0.049)	0.191 (0.036)
		4	0 (0.001)	0.022 (0.011)	0.045 (0.007)
		5**	0 (0)	0.032 (0.014)	0.027 (0.009)
		6	0.004 (0.008)	0.053 (0.025)	0.12 (0.034)
		8	0 (0)	0.082 (0.028)	0.025 (0.029)
		9	0.002 (0.002)	0.105 (0.041)	0.117 (0.035)
		11	0.053 (0.036)	0.084 (0.078)	0.274 (0.118)
		14	0.001 (0.001)	0.199 (0.088)	0.335 (0.094)
		15	0.005 (0.009)	0.469 (0.086)	0.481 (0.089)
		18	0.118 (0.016)	0.074 (0.041)	0.138 (0.037)
		20	0.025 (0.028)	0.134 (0.071)	0.222 (0.045)
		21	0.036 (0.01)	0.057 (0.046)	0.169 (0.013)
		22	0 (0)	0.055 (0.019)	0.06 (0.013)
		23	0 (0)	0.219 (0.045)	0.076 (0.02)
		25	0 (0)	0.297 (0.012)	0.011 (0.006)
		26	0.001 (0.001)	0.4 (0.119)	0.462 (0.104)
		28	0.001 (0.001)	0.342 (0.171)	0.553 (0.169)
		29	0.045 (0.051)	0.023 (0.032)	0.085 (0.046)
		30	0.004 (0.002)	0.283 (0.111)	0.399 (0.126)
		31	0.096 (0.056)	0.182 (0.168)	0.55 (0.158)
		34	0 (0)	0.082 (0.036)	0.111 (0.039)
		35	0 (0)	0.056 (0.017)	0.072 (0.031)
		36	0.102 (0.076)	0.21 (0.101)	0.279 (0.099)
		37	0.093 (0.093)	0.047 (0.052)	0.158 (0.075)
		38	0.119 (0.053)	0.032 (0.022)	0.225 (0.064)
		40	0.078 (0.053)	0.309 (0.236)	0.567 (0.214)
		41	0.07 (0.072)	0.462 (0.056)	0.433 (0.075)
2018	North Carolina	17	0.008 (0.008)	0.034 (0.028)	0.113 (0.019)
		25	0 (0)	0.284 (0.011)	0.011 (0.006)
		30	0.004 (0.004)	0.248 (0.095)	0.386 (0.104)
		44	0.042 (0.068)	0.155 (0.145)	0.266 (0.179)
		45	0.044 (0.037)	0.081 (0.101)	0.435 (0.127)
		47	0.006 (0.005)	0.222 (0.089)	0.269 (0.096)
		49	0.005 (0.009)	0.312 (0.051)	0.319 (0.06)
		51	0.009 (0.008)	0.162 (0.036)	0.328 (0.042)

		52	0.124 (0.045)	0.143 (0.03)	0.347 (0.072)
		53	0.121 (0.047)	0.049 (0.041)	0.318 (0.054)
		55	0.123 (0.06)	0.207 (0.038)	0.4 (0.055)
		56	0 (0)	0.099 (0.055)	0.116 (0.048)
		57	0.002 (0.003)	0.098 (0.035)	0.113 (0.036)
		58	0 (0)	0.051 (0.013)	0.115 (0.014)
		59	0.029 (0.025)	0.199 (0.125)	0.199 (0.118)
		60	0.11 (0.015)	0.057 (0.012)	0.209 (0.025)
		62	0.001 (0)	0.016 (0.017)	0.107 (0.019)
		63	0.082 (0.049)	0.083 (0.03)	0.063 (0.013)
		64	0 (0)	0.209 (0.081)	0.254 (0.067)
		65	0.04 (0.025)	0.163 (0.052)	0.196 (0.083)
		67	0.099 (0.14)	0.094 (0.089)	0.357 (0.202)
		71	0 (0)	0.017 (0.003)	0.002 (0.002)
		72	0.004 (0.005)	0.507 (0.169)	0.319 (0.164)
		11	0.066 (0.041)	0.026 (0.024)	0.005 (0.002)
	South Carolina	13	0.035 (0.022)	0.156 (0.092)	0.13 (0.1)
		15	0.016 (0.011)	0.087 (0.021)	0.218 (0.031)
		16	0.027 (0.014)	0.148 (0.053)	0.225 (0.026)
		17	0.153 (0.028)	0.095 (0.026)	0.11 (0.041)
		18	0.029 (0.019)	0.025 (0.018)	0.067 (0.019)
		23	0.24 (0.025)	0.103 (0.067)	0.128 (0.062)

Supplementary Table S4. Regression of relationships between proportional areas of cotton and soybean, cotton and maize, and soybean and maize within a 1-km radius of *H. zea* larval collection site.

Dependent variable	Independent variable	Slope	F (df)	P	R^2
Cotton	Soybean	NS	0,20 (1,57)	0.656	0.0035
Cotton	Maize	NS	1.59 (1,57)	0.213	0.0271
Soybean	Maize	0.3224	4.42 (1,57	0.040	0.0719
